# Increasing doses of phytase from *Citrobacter braakii* in diets with reduced inorganic phosphorus and calcium improve growth performance and lean meat of growing and finishing pigs

**DOI:** 10.1371/journal.pone.0217490

**Published:** 2019-05-24

**Authors:** Caio Abércio da Silva, Marco Aurélio Callegari, Cleandro Pazinato Dias, Ana Maria Bridi, Carlos Rodolfo Pierozan, Luciana Foppa, Claudia Cassimira da Silva Martins, Francine Taniguchi Falleiros Dias, Adsos Passos, Rafael Hermes

**Affiliations:** 1 Department of Zootechnology, Center of Agrarian Sciences, State University of Londrina, Londrina, Paraná, Brazil; 2 Akei Animal Research, Fartura, São Paulo, Brazil; 3 DSM Nutritional Products Ltda., Jaguaré, São Paulo, Brazil; University of Illinois, UNITED STATES

## Abstract

The objective of this study was to evaluate the effect of increasing doses of bacterial phytase (RONOZYME HiPhos) on performance and carcass characteristics of growing and finishing pigs. The study included 120 castrated males with initial weight of 23.21 ± 1.91 kg and 68 days of age, distributed in a randomized block design with five treatments and eight replicates with three animals each. The pigs were fed five corn-soybean meal-based diets: positive control (PC), supplemented with inorganic phosphorus and calcium; negative control (NC), with 0.13% reduction in available phosphorus and 0.11% in calcium; and three NC diets supplemented with 1,000, 2,000, and 3,000 phytase units (FYT)/kg in the feed. Compared with the NC diets without phytase, diets with 1,000, 2,000, and 3,000 FYT/kg inclusion increased the daily weight gain by +12% (quadratic, p<0.05) during the growing I period; +2.9, +2.9, and +10.5% (linear, p<0.01), respectively, during the growing II period; and +4.1, +5.1, and +8.2% (linear, p<0.001), respectively, over the entire experimental period. The daily feed intake increased by 0, +2.8, and +4.3% (linear, p<0.05), respectively, considering the entire experimental period; and the final live weight increased by +3.2, +4.2, and +6.1% (linear, p<0.001), respectively. The phytase treatments did not influence feed conversion ratio, carcass weight and yield, backfat thickness, loin depth and carcass lean meat. According to the European Carcass Classification (SEUROP), however, the animals fed the PC diet and the three phytase levels had more carcasses classified as E (between 55–60% lean meat) when compared to carcasses of pigs fed the NC. Supplementing increasing levels of phytase to a corn- and soybean meal-based diet with inorganic P and Ca reduction improved daily weight gain and feed intake of growing pigs, and such effects were maintained until slaughter age.

## Introduction

Phytases of bacterial origin are the exogenous enzymes most widely used in the diets of monogastric animals, acting on the hydrolysis of phytate (myo-inositol 1,2,3,4,5,6-hexakis [dihydrogen] phosphate) to release the phosphate from this complex [1,2]. Phytases are commonly developed by means of genetic engineering [3], such as phytase from *Escherichia coli* expressed in *Trichoderma reesei* and phytase from *Citrobacter braakii* expressed in *Aspergillus oryzae* [2]. They are classified in two groups, 3-phytases or 6-phytases, according to the site in the phytate molecule where the first orthophosphate is hydrolyzed [4,5].

Phytase is usually added to pig diets at 500 FYT/kg [6,7], but, at this level, less than 50% of the phytate in the diet is commonly hydrolyzed. Therefore, higher inclusion levels are necessary in order to hydrolyze more than 60% of the phytate, reducing its antinutritional effects and improving efficiency of organic phosphorus utilization [2]. The so-called “extra phosphoric” effects of phytase should also be taken into consideration as they increase the digestibility of calcium, amino acids, and even energy [8–11]. Thus, the levels of these nutrients in the feeds can be decreased.

Considering that feed costs can reach up to 60–70% of the total cost of pig production [7], increasing the conventional dose of phytase by 1.5 to 2 times can be economically attractive when the value of nutrients being released is compared to the cost of the phytase being used [12]. Its efficacy, however, depends on several factors such as animal category, type of diet, and the phytase source [4]. Thus, increasing the enzyme inclusion level does not necessarily represent a linear improvement in nutrient utilization.

According to Adeola and Cowieson [1], a phytase inclusion level exceeding 2,500 FYT/kg feed characterizes a high dose of phytase inclusion. There are many reports in the literature stating that high doses of phytase inclusion improve the performance of weaned piglets. Gonçalves et al. [13], however, observed that the effects in finishing pigs are variable and appear to be more evident if the concentrations of digestible phosphorus and other nutrients are marginal in the diet. The benefits of this inclusion pattern are related not only to animal performance, but also to health, well-being, and, possibly, to meat quality [14], the latter being determined by the significant increase in the plasma levels of myo-inositol [15]. Besides that, the levels of inorganic phosphorus in animal diets can be decreased by using phytase, thus reducing phosphorus excretion to the environment [16].

In line with the optimization of exogenous phytase effects when its levels are increased in the diet, another concept has been postulated in recent years, i.e., “phytate-free nutrition.” This concept is being valued as it includes nutritional aspects such as better use of phosphorus, the extra-phosphoric effects, and the complete dephosphorylation of phytate into myo-inositol [17]. Phytases, however, are not able to achieve complete dephosphorylation of phytate as it contains a refractory axially-aligned phosphate group on carbon 2 on the myo-inositol ring that is incompatible with most commercially available exogenous phytases [18], which requires a cooperative effort from endogenous phosphatases and other potentiating enzymes.

The efficacy of high doses of phytase from *Citrobacter braakii* expressed in *Aspergillus oryzae* has already been tested on the performance of nursery piglets [19] and growing and finishing [20] in diets including barley, wheat and oats but not in diets based only on corn and soybean meal. Therefore, the objective of the present trial was to evaluate the effect of increasing doses of this microbial phytase (RONOZYME HiPhos, DSM Nutritional Products, Brazil) on performance and carcass characteristics of growing and finishing pigs using corn- and soybean meal-based feeds.

## Material and methods

This study was carried out in strict accordance with the recommendations of the Guide for the Care and Use of Laboratory Animals of the National Animal Experimentation Control Council of Brazil (CEUA). The trial was approved by the Ethics Committee of Animal Experiments of Akei Animal Research (protocol number: 003.2016). The animals were obtained from a commercial farrow-to-finish farm after they left the nursery phase and were housed in a barn with 40 pens (5.85 m^2^/pen) equipped with a Dutch-model feeder, nipple drinking trough, and partially slatted concrete floor. Thermal control of the barn was manual by managing the curtains; temperature and relative humidity were recorded by an Instrutemp ITLOG 80 datalogger. Maximum and minimum air temperature and relative humidity during the experimental period were 21.16 ± 1.20°C and 29.71 ± 2.44°C, and 66.92 ± 14.91%, respectively.

The study included a total of 120 castrated males (Camborough x PIC 337) with initial weight of 23.21 ± 1.91 kg and 68 days of age and employed a randomized block design (based on initial weight) with five treatments and eight replicates. Each pen contained three animals and served as the experimental unit. The treatments included five diets: positive control (PC), supplemented with inorganic phosphorus (P) and calcium (Ca) provided by dicalcium phosphate and limestone; negative control (NC), with reduction in available phosphorus (-0.13%) and calcium (-0.11%); 1,000 phytase units (FYT) Diet, the NC diet supplemented with 1,000 FYT/kg feed; 2.000 FYT Diet, the NC diet supplemented with 2,000 FYT/kg feed; and 3,000 FYT/kg feed, characterized as “high dose.” RONOZYME HiPhos (DSM Nutritional Products, Brazil) was the phytase used in the experiment, a 6-phytase produced by introducing synthetic genes that simulate a phytase gene from *C*. *braakii* ATCC 51113 and express the coded phytase product in *A*. *oryzae* [19]. A FYT was defined as the amount of enzyme needed to release 1 μmol of inorganic phosphorus from sodium phytate per minute at 37°C [3]. Phytase was added to the experimental diets as an additive at 1.0, 2.0, and 3.0 kg/ton feed.

The animals were submitted to a feeding program with four phases: growing I (68–91 days of age); growing II (92–112 days of age); finishing I (113–140 days of age); and finishing II (141–156 days of age) (Table 1). All feeds were based on corn and soybean meal and were mashed and formulated according to the requirements of the Brazilian Tables for Poultry and Swine [21]. Both feed and water were provided *ad libitum*.

**Table 1:** Composition (inclusion %) of the diets used during the experimental period^a^.

	Growing I^b^		Growing II^c^		Finishing I^d^		Finishing II^e^	
	PC	NC	PC	NC	PC	NC	PC	NC
Ingredient								
Corn kernels	68.54	69.65	72.46	73.45	77.09	77.77	81.76	82.42
Soybean meal	28.10	27.90	24.72	24.54	20.39	20.27	15.90	15.79
Dicalcium phosphate	1.14	0.44	0.93	0.22	0.86	0.16	0.79	0.09
Limestone	0.70	0.86	0.64	0.80	0.60	0.76	0.56	0.73
Soybean oil	0.58	0.21	0.27	0.00	0.02	0.00	0.00	0.00
Lysine	0.12	0.12	0.15	0.15	0.21	0.21	0.24	0.24
Methionine	0.03	0.03	0.02	0.02	0.03	0.03	0.02	0.02
Threonine	0.02	0.02	0.03	0.03	0.03	0.03	0.03	0.03
Tryptophan	0.00	0.00	0.00	0.00	0.00	0.00	0.02	0.02
Vitamin premix^f^	0.10	0.10	0.10	0.10	0.10	0.10	0.08	0.08
Mineral premix^g^	0.10	0.10	0.10	0.10	0.10	0.10	0.10	0.10
Salt	0.40	0.40	0.40	0.40	0.40	0.40	0.30	0.30
Adsorbent	0.15	0.15	0.15	0.15	0.15	0.15	0.15	0.15
Inert (kaolin)	0.03	0.03	0.03	0.03	0.03	0.03	0.03	0.03
Calculated composition								
Metabolizable energy (kcal/kg)	3,230	3,230	3,230	3,230	3,230	3,230	3,230	3,230
Crude protein	18.25	18.25	17.07	17.07	15.53	15.53	13.92	13.92
Ca	0.64	0.53	0.55	0.44	0.51	0.40	0.47	0.36
P, total	0.52	0.40	0.47	0.35	0.45	0.32	0.43	0.30
P, available	0.31	0.18	0.27	0.14	0.25	0.12	0.23	0.10
Ca:P, available	2.06	2.94	2.04	3.14	2.04	3.33	2.04	3.60
Digestible lysine	0.94	0.94	0.89	0.89	0.83	0.83	0.75	0.75
Digestible methionine	0.28	0.28	0.27	0.27	0.26	0.26	0.23	0.23
Digestible methionine + cysteine	0.54	0.54	0.51	0.51	0.48	0.48	0.44	0.44
Digestible threonine	0.64	0.64	0.61	0.61	0.56	0.56	0.50	0.50
Digestible tryptophan	0.20	0.20	0.18	0.18	0.16	0.16	0.15	0.15
Sodium	0.18	0.18	0.18	0.18	0.18	0.18	0.18	0.18

PC = positive control. NC = negative control.

^a^Phytase was added to the negative control diets at 1,000, 2,000, and 3,000 phytase units (FYT) per kg of feed in the four evaluation periods.

^b^68 to 91 days of age.

^c^92 to 112 days of age.

^d^113 to 140 days of age.

^e^141 to 156 days of age.

^f^Vitamin premix provided per kg of diet: 6,000 IU vitamin A; 1,500 IU vitamin D_3_; 15 mg vitamin E; 1.5 mg vitamin K_3_; 1.35 mg vitamin B_1_; 4 mg vitamin B_2_; 2 mg vitamin B_6_; 20 μg vitamin B_12_; 20 mg; 9.35 mg pantothenic acid; 600 μg folic acid; 80 μg biotin; 300 μg Se.

^g^Mineral premix provided per kg of diet: 100 mg Fe; 10 mg Cu; 40 g Mn; 1 mg Co; 100 mg Zn; 1.5 mg I.

Performance variables of daily weight gain (DWG), daily feed intake (DFI), and feed conversion (FCR) were evaluated at the beginning of the study and at the end of each phase. At 156 days of age, all animals were taken to a commercial packing plant for slaughter. Solid foods were withdrawn 12 h before transportation while water was available until slaughter. According to current legislation, the animals were desensitized using a Petrovina IS 2000 stunning device with two electrodes (350 V and 1.3 A) for approximately 3 s and then bled by severing the large vessels in the neck. After slaughter, scalding, and evisceration, carcasses were divided longitudinally and refrigerated at 2 ± 1°C for 24 h in a cooling chamber. Each carcass underwent electronic evaluation (Hennessy Grade Probe, Hennessy Grading Systems, Auckland, NZ) by trained personnel. The recorded variables were: carcass weight (kg); carcass yield (%); backfat thickness (mm) measured at point P2, i.e., 59 mm lateral to the carcass dorsal midline immediately caudal to the last rib on one of the carcass sides; loin depth (mm) measured at point P2; carcass lean meat percentage (%), and carcass lean meat yield (kg), obtained by multiplying the carcass weight by the lean meat percentage. Lean meat yield was also calculated according to the European Carcass Classification, where letters S, E, U, R, O, and P represent the following lean meat percentages on the carcass: >60, 55–60, 50–55, 45–50, 40–45, and <40, respectively. Feces were classified as normal, pasty, soft, and diarrheic [22] in the two first weeks of the experimental period. The diarrhea score was calculated by dividing the number of days the animals had diarrhea by the total number of days evaluated.

Pen served as the experimental unit for performance and carcass data. The data were submitted to analysis of variance (ANOVA) and means were compared by Tukey’s test. Non-parametric data were analyzed by Chi-square test. In both tests, the significance level for the difference between means, α, was determined to be 0.05. Additionally, all performance and carcass parameters were submitted to regression analysis at 0 (negative control), 1,000, 2,000, and 3,000 FYT/kg feed levels. The statistical program R version 3.5.0 was used for all statistical analysis.

## Results

A quadratic effect (p<0.05) for DWG and weigh at 91 days of age was found in the growing I (68 to 91 days of age) phase (Table 2). There were no differences in the results for these two performance parameters when animals in the PC group were compared to animals fed diets with phytase.

**Table 2:** Mean live weight (LW), daily weight gain (DWG), daily feed intake (DFI), and feed conversion rate (FCR) during each phase and total evaluation period according to the experimental treatment.

Variable	Treatment^a^					CV	P Value	P Value (R^2^)^b^	
	PC	NC	1,000 FYT	2,000 FYT	3,000 FYT			Linear	Quadratic
	Growing I (68–91 days of age)								
LW68 (kg)	23.23	23.21	23.21	23.23	23.23	0.18	0.768	NS	NS
DWG (kg)	0.92a	0.83b	0.93a	0.93a	0.93a	7.25	0.016	-	0.047 (0.93)^c^
DFI (kg)	1.76	1.68	1.74	1.68	1.81	13.83	0.798	NS	NS
FCR (kg/kg)	1.91	2.03	1.89	1.81	1.94	12.43	0.466	NS	NS
	Growing II (92–112 days of age)								
LW91 (kg)	44.49a	42.28b	44.62a	44.62a	44.58a	3.42	0.015	-	0.046 (0.93)^d^
DWG (kg)	1.11ab	1.05b	1.08ab	1.08ab	1.16a	6.38	0.047	0.007 (0.78)^e^	-
DFI (kg)	2.56	2.49	2.49	2.49	2.65	7.59	0.397	NS	NS
FCR (kg/kg)	2.33	2.37	2.32	2.31	2.29	8.08	0.942	NS	NS
	Finishing I (113–140 days of age)								
LW112 (kg)	67.73a	64.39b	67.25a	67.95a	68.95a	2.90	0.001	<0.001 (0.90)^f^	-
DWG (kg)	1.01	1.00	1.07	1.04	1.08	7.14	0.164	NS	NS
DFI (kg)	2.95	2.90	2.93	2.94	3.05	6.08	0.486	NS	NS
FCR (kg/kg)	2.94	2.89	2.74	2.84	2.82	6.48	0.259	NS	NS
	Finishing II (141–156 days of age)								
LW140 (kg)	96.04ab	92.51b	97.25a	96.92a	99.23a	5.15	<0.001	0.015 (0.15)^g^	NS
DWG (kg)	1.05	1.06	0.99	1.08	1.06	11.97	0.618	NS	NS
DFI (kg)	3.17	3.20	3.08	3.18	3.18	8.30	0.917	NS	NS
FCR (kg/kg)	3.07	3.01	3.14	2.98	3.00	8.23	0.680	NS	NS
LW156 (kg)	112.84ab	109.53b	113.01ab	114.17ab	116.20a	3.49	0.035	<0.001 (0.96)^h^	-
	Period total (68–156 days of age)								
DWG (kg)	1.02ab	0.98b	1.02ab	1.03ab	1.06a	4.40	0.036	<0.001 (0.95)^i^	-
DFI (kg)	2.59	2.54	2.54	2.61	2.65	3.94	0.142	0.026 (0.92)^j^	-
FCR (kg/kg)	2.55	2.59	2.50	2.52	2.51	4.70	0.590	NS	NS

PC = positive control. NC = negative control. FYT = phytase units per kg of feed. CV = coefficient of variation. NS = not significant.

^a^Means followed by different letters on the same row are significantly different by Tukey’s test at 5%.

^b^Linear and quadratic response for increasing phytase levels in the diet. R^2^ represents the coefficient of determination.

^c^Y = 0.834103+0.107518x-0.025905x^2^; P value = 0.04709; R^2^ = 0.93

^d^Y = 42.3904+2.48312x-0.59687x^2;^ P value = 0.0460; R^2^ = 0.93

^e^Y = 1.04382+0.0321032x; P value = 0.00716; R^2^ = 0.78

^f^Y = 64.9781+0.143739x; P value = 0.00035; R^2^ = 0.90

^g^Y = 93.5000+0,001983x; P value = 0.0156; R^2^ = 0.15

^h^Y = 110.055+2.11542x; P value = 0.00048; R^2^ = 0.96

^i^Y = 0.986913+0.0239583X; P value = 0.00051; R^2^ = 0.95

^j^Y = 2.52177+0.0424053; P value = 0.0264; R^2^ = 0.92

Daily weight gain was worse in the NC treatment in the growing II (92–112 days of age) phase compared to the group receiving 3,000 FYT/kg feed, which had the best results (p<0.05), while intermediate results were obtained for the treatments with the inclusion of 1,000 and 2,000 FYT/kg. At 112 days, pigs fed diets containing phytase had higher live weight (LW) when compared to the NC group. It was also found that inclusion of phytase had a positive linear regression effect (p<0.01) on DWG and LW.

In the finishing I (113–140 days of age) and II (141–156 days of age) phases, no treatment had a significant effect on DWG, but at 140 days of age the pigs that received phytase showed a better LW when compared to the NC group, and inclusion of phytase demonstrated a positive linear effect (p<0.05) on this parameter. At 156 days of age, pigs in treatment NC had worse LW when compared to the treatment with 3,000 FYT/kg (p<0.05), while intermediate LW values were found for PC and animals in treatments with 1,000 and 2,000 FYT/kg feed inclusion levels. Final LWs increased, by +3.2, +4.2, and +6.1%, respectively, compared to the NC treatment, when 1,000, 2,000, and 3,000 FYT/kg feed were added to the NC diets, however, only the increase obtained with the inclusion of 3,000 FYT/kg feed was significant. Results indicated that increasing levels of phytase had a positive linear effect (p<0.001) on final LW.

The same result was found for DWG over the experimental period (68–156 days of age). Pigs of the NC treatment had the worst DWG; the group treated with 3,000 FYT/kg feed had the best DWG (p<0.05), and the other groups had intermediate values. Compared to the NC group, supplementation with 1,000, 2,000, and 3,000 FYT/kg feed increased DWG by +4.1, +5.1, and +8.2%, respectively. However, only the increase in DWG obtained with the inclusion of 3,000 FYT/kg feed over the negative control was significant. A positive linear effect (p<0.001) on DWG was also seen with the inclusion of increasing levels of phytase.

A positive linear regression effect (p<0.05) for DFI indicated higher feed intake over the evaluation period when phytase supplementation increased. No regression effect or differences among treatments were found for FCR, mortality, or diarrhea. No differences or regression effects were found among treatments for all carcass characteristics evaluated (Table 3).

**Table 3:** Means of carcass yield, backfat thickness, loin depth, and lean meat percentage and yield according to the experimental treatment.

Variable	Treatment^a^					CV	P value	P value (R^2^)^b^
	PC	NC	1,000 FYT	2,000 FYT	3,000 FYT			
Carcass weight (kg)	80.00	80.02	82.29	81.66	82.40	4.25	0.451	NS
Carcass yield (%)	71.00	72.85	72.90	71.89	70.93	4.39	0.576	NS
Backfat thickness (mm)	16.15	17.70	18.00	17.42	17.37	10.15	0.299	NS
Loin depth (mm)	60.80	64.28	63.10	60.98	62.40	4.97	0.159	NS
Lean meat (%)	55.14	54.30	53.96	54.18	54.37	2.51	0.506	NS
Lean meat (kg)	44.05	43.44	44.37	44.21	44.79	5.37	0.846	NS

PC = positive control. NC = negative control. FYT = phytase units. CV = coefficient of variation. NS = not significant.

^a^Means followed by different letters on the same row are significantly different by Tukey’s test at 5%.

^b^Linear response to increasing phytase levels in the diet. R^2^ represents the coefficient of determination.

Considering the results of the European Carcass Classification standard (Table 4), most carcasses evaluated had E classification (55–60% lean meat). Animals fed PC diets with the three phytase inclusion levels had more carcasses in this classification than pigs fed the NC diet (p<0.05). At both classification extremes, pigs fed the PC diets with the three phytase inclusion levels had fewer carcasses with >60% (classification S) and 50–55% (classification U) lean meat when compared to animals fed NC diets (p<0.05). No carcass had less than 50% lean meat.

**Table 4:** Effect of treatments on the number of carcasses categorized by lean meat yield according to the European Carcass Classification.

Variable	Treatment^a^				
	Positive control	Negative control	1,000 FYT	2,000 FTY	3,000 FYT
Class S^**b**^	4b	6a	2b	2b	3b
Class E^**b**^	19a	12b	20a	18a	19a
Class U^**b**^	1b	6a	2b	3b	2b

^a^Absolute numbers followed by different letters on the same row indicate statistical differences between treatments by the Chi-square test at 5%.

^b^S, E, and U represent, respectively, >60%, 55–60%, and 50–55% lean meat on the carcass.

## Discussion

The DWG of animals fed the diet without phytase (NC) was lower (p<0.05) when compared to PC only in the growing I and II phases. This difference, however, was not maintained in the next phases (finishing I and II). Braña et al. [5] also reported that castrated pigs fed diets with low P levels and no phytase had lower DWG than animals fed recommended P levels during the growing phase, but this difference remained during the finishing phase. While in the present study the diet formulations have followed the recommendation by Rostagno [21], ensuring non-variable reductions in P and Ca in all NC diets, Braña et al. [5] used formulations based on the NRC [23] and, in the finishing period, the reduction in P in the NC diet compared to the PC one was higher than in the present study (-60.0 *versus* -52.2%, respectively). In addition, no reduction in Ca was found in the NC diets of that study, which led to excessive increase in the available Ca:P ratio, intensifying the negative effect of deficiency in available P in the NC diets.

The data in this study were different from the findings by Brady et al. [24] and Madrid et al. [25]. Working with growing pigs, they did not observe DWG differences between negative (low P, no phytase) and positive treatments. However, their experimental feeds had smaller differences in P level and Ca:P ratio between negative and positive diets compared with the present study. A multivalent cation, Ca increases the formation of phytin crystals, which are insoluble and restrict the access of phytase to phytate and, therefore, P availability [26].

The highest phytase inclusion levels increased pig DWG and LW when compared to the NC diet, mainly in the first phases. The treatment with 3,000 FYT/kg feed showed the best and longest lasting responses for these parameters. The positive linear effect observed up to the 3,000 FYT/kg feed limit indicates that higher levels can further improve these results.

Based on these comments, there are two possible factors involved in our results. First, the not balanced Ca:P ratio between the PC and NC, which PC has lower Ca:P than NC, might have resulted in the decrease performance in NC, as related Silva et al. [26]. Another possibility is the phytase improving performance by restoring the Ca: available P ratio, especially for promoting a greater P releasing compared with Ca, as demonstrated by Almeida et al. [3]. These authors worked with six treatments (positive control, negative control, 500, 1,000, 2,000 and 4,000 FYT/kg) and observed a progressive improvement on Ca and P digestibility, which P digestibility was around three times greater than Ca digestibility.

Several studies have confirmed the DWG increase when different phytases have been used during the growing phase, such as those originated from *Aspergillus niger* (1,000 FYT/kg feed) [27] and from *Peniophora lycii* (at 250, 500, and 1,000 FYT/kg feed doses, resulting in a quadratic effect) [24]. In addition, during growing and finishing, the phytase originated from *Schizosaccharomyces pombe* [5] led to a linear effect when used at 250, 500, 750, 1,000, and 10,000 FYT/kg feed doses. The effect of phytase (1,000, 2,000, and 3,000 units/kg feed) used in the present trial was previously evaluated in diets with other ingredients (corn, soybean meal, barley, and oat meal) [20]. Those authors verified that phytase treatments promoted a consistent increase in DWG of growing and finishing pigs (70–154 days of age) in two consecutive trials. Its efficacy on P and Ca digestibility was also shown to be positive when added to diets in the nursery and growing phases [3,19,28]. Moreover, performance improved when phytase was added to diets based on wheat, barley, corn, and soybean fed to nursery piglets at doses from 1,000 to 4,000 FYT/kg feed [19].

Unlike P and Ca digestibility, which is effectively increased by phytase in pigs [3,24,25], studies on protein and amino acid digestibility in growing and finishing pigs had conflicting and inconclusive results [29–31]. According to Cowieson et al. [17], diets that do not provide sufficient macro- and micro-nutrients (due to formulation or high levels of enzyme matrix) can prevent the pathways stimulated by myo-inositol from improving FCR by increasing protein accumulation and downregulating gluconeogenesis. Cowieson et al. [15] and Schmeisser et al. [32] indicated a positive effect of phytase on the concentration of myo-inositol, whose action mimics insulin, with similar effects on GLUT 4 when blood glucose concentration increases, which is involved in aspects related to gluconeogenesis and improves digestibility, weight gain, and feed conversion.

The growth improvement caused by phytase can be attributed to the better utilization of plant-origin P and to the release of starch and protein bound to phytic acid [24]. Ketaren et al. [27] found an increase in protein accretion in female pigs supplemented with phytase (1,000 FYT/kg feed) during 35 days of the growing period, suggesting that the enzyme contributes to increasing the utilization of protein from soybean meal. Other authors, however, reported that the inclusion of 500 or 1,000 FYT/kg feed did not result in significant differences in performance or protein deposition in both females and castrated males during the growing phase [33,34].

In this study, based on some evaluations [19,20,35], the presumed value of the phytase matrix, at 1,000 FYT/kg of feed, was to release 0.13 and 0.11% more available P and total Ca from the feed, but the hypothesized extra phosphoric effect observed in our results are based on founds of other studies. Considering exclusively the effects on dietary Ca and P, when high levels of phytase were used, Guggenbuhl et al. [35] found a progressive increase of total apparent digestibility (ATTD) of Ca and P to 4,000 FTY/kg feed for piglets during the nursery phase and the same results for 2,000 FYT/kg for growing pigs. On the other hand, Guggenbuhl et al. [36], working with growing pigs fed with corn-soybean meal-barley based diet, observed, compared with NC diet, best Ca and P ATTD (p<0.05) when was used phytase (RONOZYME HiPhos) 500 FTY/kg feed, but at 1,000 FYT/kg, besides Ca an P digestibility, the protein, total amino acids and essential amino acids digestibility was improved. Velayudhan et al. [37], working with growing and finishing pigs fed with corn-soybean meal based diet supplemented by phytase (250, 500, 1,000 and 2,000 FTY/kg), verified a linear increased (p<0.05) of crude matter, crude protein and energy ATTD of diets.

Microbial phytases can have distinct origins, which may explain differences in their bio-efficacy per unit of phytase analyzed [8]. The inclusion strategy for these enzymes should vary depending on their concentration in the diet and on whether the extra-phosphoric effects are the priority instead of phosphorus availability [17]. According to Selle and Ravindran [10], phytase inclusion rates should be based on the substrate (phytate) levels in the diet and not on the enzyme inclusion rate. It is possible that low levels of phytate in the diet require phytase inclusion to be higher to overcome low concentrations of the substrate. Smaller differences in results can be obtained between low and high phytase inclusion levels under intermediate levels of phytate in the diet, in contrast with high phytate levels, when the responses can be perceived with standard phytase inclusion [17].

In the present study, the inclusion of phytase during the finishing periods did not improve performance when compared to the control diets, even at 3,000 FYT/kg feed. Thus, the results were similar to those found by Dersjant-Li et al. [7] when working with pigs in the finishing (85–125 kg LW) using a 6-phytase from *Buttiauxella* sp. (250, 500, and 1,000 FYT/kg feed). According to Cowieson et al. [17], animal genetics and age are among the fundamental factors to be taken into consideration to optimize the strategies of extra-phosphoric effects of phytase. Thus, the absence of differences in DWG among treatments in the finishing period could be attributed to the fact that the animals are close to their maximum genetic potential for weight gain. Another hypothesis is that the phytase levels among treatments have reached a balance due to endogenous phytase production by the pigs since phytase concentration in the intestinal mucosa increases with age, which improves the ability by the animals to use phytic acid in the diet [25]. Finally, as previously discussed, the performance of pigs fed NC diets during the finishing phase was comparable to that of animals fed PC diets. Therefore, the reduction in available P in the NC diets may not have been sufficient for the addition of phytase to cause any improvement in performance.

No differences were found among treatments for DFI when each period was analyzed separately. Similar results were also reported by Braña et al. [5] in two observations, one in the growing period and one in finishing. However, when the total experimental period was analyzed in the present study, a positive linear response (p<0.05) was found for this parameter according to the increase in phytase inclusion, which can be explained by the accumulation of DFI differences in each period and the positive linear response for body weight.

Although no regression effects or statistical difference were found for FCR, phytase treatments numerically improved the parameter until the end of the finishing I period. Guggenbuhl et al. [19], when including nursery piglets in their study with the same phytase, found that FCR improved when the inclusion level was 2,000 FYT/kg feed in comparison with the negative control, positive control, and 500 FYT/kg feed inclusion level. Results with 1,000 and 4,000 FYT/kg feed doses were intermediate. In other studies, with other phytases, FCR improved during growing and/or finishing [5,27], a response attributed to higher protein deposition rates due to the additional P released by the enzyme [5].

No differences on carcass characteristics were found among treatments, nor any regression effects. Similar results were reported by Dersjant-Li et al. [7] when growing and finishing diets of pigs were supplemented with 6-phytase from *Buttiauxella* sp. expressed in *Trichoderma reesei* (250, 500, and 1,000 FYT/kg feed), and also found by Fandrejewski et al. [33], who used a 3-phytase originated from and expressed in *Aspergillus niger* (1,000 FYT/kg feed) during the growing period. However, Lozano et al. [38], when working with *Aspergillus niger* phytase supplementation of finishing pigs, observed greater loin depth with 500 and 1,000 FYT/kg feed compared to the control group.

On the other hand, Brady et al. [24] reported a linear increase in backfat thickness and a linear decrease in lean meat content with the use of a phytase from *Peniophora lycii* (500, 750, and 1,000 FYT/kg feed). Those authors attribute these effects to a progressive utilization of energy from the diet generated by the increasing doses of phytase.

In some studies, the use of phytase showed inconsistent results in energy utilization [39] and affected carcass characteristics. In the present study, diets with phytase did not increase fat deposition, although DFI and DWG did increase. This result suggests that the “additional” energy provided using phytase was not sufficient to promote fat deposition. At the same time, common phytase variations should also be considered and effects are attributed to the phytase origin and expression, inclusion level, and other inherent characteristics of the diet.

Animals fed the NC diet had more carcasses with higher lean meat content (classified as S; >60% lean meat). However, the percentage of carcasses in this classification corresponds to only 25% (six carcasses) of all carcasses in the NC group. Carcasses classified as E (55–60% lean meat) represented 73.9% (88 of 119) of all evaluated carcasses. The NC group represented 13.6% of this total, while the PC group and those supplemented with 1,000, 2,000, and 3,000 FYT/kg feed represented 21.6%, 22.7%, 20.5%, and 21.6%, respectively. Thus, it is possible to consider that carcasses from the PC and from the groups with phytase inclusion had better classification for lean meat percentage, as well as better uniformity standard compared with NC.

Phosphorus is essential in the formation and mineralization of the bone organic matrix and in muscle development [40]. Pigs of high genetic potential for meat deposition have high lean meat content [41], which may increase the demand for available phosphorus [40]. Thus, when the minimum requirement of this mineral is supplied, in this case, by phytase, more animals have the opportunity of showing their genetic potential for lean tissue deposition, up to a certain limit, thus improving carcass uniformity. Associated with previously reported results for carcass characteristics, particularly lean meat content, the safety of high phytase inclusion level of 3,000 FYT/kg feed is established.

## Conclusion

Supplementation with phytase at 1000, 2000, or 3000 FYT/kg feed in diets based on corn and soybean meal, with reduced inorganic phosphorus and calcium, improved daily weight gain and feed intake of growing pigs with permanent results until slaughter age with a dose-response relationship. Moreover, the supplementation of phytase may support the reduction of calcium and phosphorus in the diet without impairment of lean meat traits.

## Supporting information

S1 File Table 2 data is in S1 File. (XLS)

S2 File Table 3 and Table 4 data is in S2 File. (XLS)
